# Site Specialization of Human Oral *Veillonella* Species

**DOI:** 10.1128/spectrum.04042-22

**Published:** 2023-01-25

**Authors:** Jonathan J. Giacomini, Julian Torres-Morales, Floyd E. Dewhirst, Gary G. Borisy, Jessica L. Mark Welch

**Affiliations:** a The Forsyth Institute, Cambridge, Massachusetts, USA; b Harvard School of Dental Medicine, Boston, Massachusetts, USA; c Marine Biological Laboratory, Woods Hole, Massachusetts, USA; Ohio State University

**Keywords:** *Veillonella*, pangenomics, metagenomics, metapangenomics, tropism, site specialists, niche partitioning

## Abstract

*Veillonella* species are abundant members of the human oral microbiome with multiple interspecies commensal relationships. Examining the distribution patterns of *Veillonella* species across the oral cavity is fundamental to understanding their oral ecology. In this study, we used a combination of pangenomic analysis and oral metagenomic information to clarify *Veillonella* taxonomy and to test the site specialist hypothesis for the *Veillonella* genus, which contends that most oral bacterial species are adapted to live at specific oral sites. Using isolate genome sequences combined with shotgun metagenomic sequence data, we showed that *Veillonella* species have clear, differential site specificity: Veillonella parvula showed strong preference for supra- and subgingival plaque, while closely related V. dispar, as well as more distantly related V. atypica, preferred the tongue dorsum, tonsils, throat, and hard palate. In addition, the provisionally named *Veillonella* sp. Human Microbial Taxon 780 showed strong site specificity for keratinized gingiva. Using comparative genomic analysis, we identified genes associated with thiamine biosynthesis and the reductive pentose phosphate cycle that may enable *Veillonella* species to occupy their respective habitats.

**IMPORTANCE** Understanding the microbial ecology of the mouth is fundamental for understanding human physiology. In this study, metapangenomics demonstrated that different *Veillonella* species have clear ecological preferences in the oral cavity of healthy humans, validating the site specialist hypothesis. Furthermore, the gene pool of different *Veillonella* species was found to be reflective of their ecology, illuminating the potential role of vitamins and carbohydrates in determining *Veillonella* distribution patterns and interspecies interactions.

## INTRODUCTION

The genus *Veillonella* is comprised of anaerobic, Gram-negative cocci (1, 2) that are abundant in the human oral cavity. Their physiology is characterized by active fermentation of organic acids, primarily lactic acid, and inability to use carbohydrates or amino acids as an energy source (3, 4). Because of their ability to consume lactic acid, *Veillonella* organisms are thought to establish commensal relationships with Streptococcus and other organisms that generate lactic acid from the metabolism of carbohydrates (5, 6). Despite the potential importance of the *Veillonella* genus for taxon-taxon interaction, little is known about the genetic variation between species or strains that may be critical for adaptation of *Veillonella* to different niches within the mouth. An understanding of oral *Veillonella* ecology requires delving deeper into the genomic differences that underlie physiological differences between individual species in the sites they inhabit.

In a recent review of the literature (7), we advanced the site specialist hypothesis of the oral microbiome to summarize the evidence that separate sites in the mouth, such as the teeth, tongue dorsum, and buccal mucosa, while bathed in the same saliva, are profoundly different in the microbiotas they support. These differences consist not merely of shifts in the proportions of the same taxa but of colonization by substantially different taxa. Although most genera of oral bacteria are represented throughout the human oral cavity, many species have strong tropisms for specific sites in the mouth (8). To date, support for the site specialist hypothesis is based primarily on 16S rRNA gene sequence data (8–10) as well as older studies with cultivated organisms (11, 12). These approaches have limitations, as 16S rRNA gene phylogenies assign species designations based on a small region of a single gene, and cultivation-based studies examine only a few species. A broader basis is necessary for evaluating the site specialist hypothesis.

Currently, taxonomic assignment of species within the genus *Veillonella* rests on multiple marker genes. Seven species of human oral *Veillonella*, 5 named and 2 unnamed, are currently recognized in the expanded Human Oral Microbiome Database (eHOMD; http://www.homd.org) (13, 14), and 11 more species are recognized in the List of Prokaryotic Names with Standing in Nomenclature (15), 6 of which are human and 5 of which are of animal origin. Although membership in the genus *Veillonella* can be determined with high certainty from 16S rRNA gene sequences, discrimination between some of the members at the species level has been problematic because of low dissimilarity between 16S rRNA genes of different species (16, 17) and because of heterogeneity of the four 16S rRNA gene copies within a single cell (18). These difficulties have led investigators to introduce the use of additional marker gene sequences (19) to discriminate species.

Circumvention of these limitations, resolution of taxonomic uncertainties, and insight into the gene drivers of niche adaptation can be attained by a “metapangenomics” approach (20), which brings together pangenome analysis and environmental metagenomic information. The pangenome, the sum of all genes found across members of a given group, reveals the functional essence and diversity held within that group (21, 22). Although the pangenome contains the theoretical palette of traits available, by itself it does not say which are actually realized in a particular environment. Complementary to the pangenome is whole-genome shotgun metagenomic information obtained from environmental samples. Mapping short-read metagenomic data to the pangenome makes it possible to estimate the relative abundance of environmental populations without the limitations of cultivation or assembly (23–25). This combination of metagenomes and pangenomes can demonstrate the distribution of taxa across environmental habitats, thus indicating environment-specific selection pressures. Functional annotation can suggest genes putatively required for site adaptation.

In this study, we used a metapangenomics approach to assess the distribution of natural *Veillonella* populations in the healthy human oral cavity and identify genes that characterize human and oral *Veillonella* species. We showed that *Veillonella* species have clear, differential site specificities, validating the site specialist hypothesis. We then identified specific genes associated with key functions that may facilitate habitat specialization for different *Veillonella* species. Our findings provide a first step for identifying the molecular underpinnings that drive *Veillonella* distribution in the oral microbiome.

## RESULTS

### *Veillonella* pangenome, phylogeny, and ANI.

**FIG 1:**
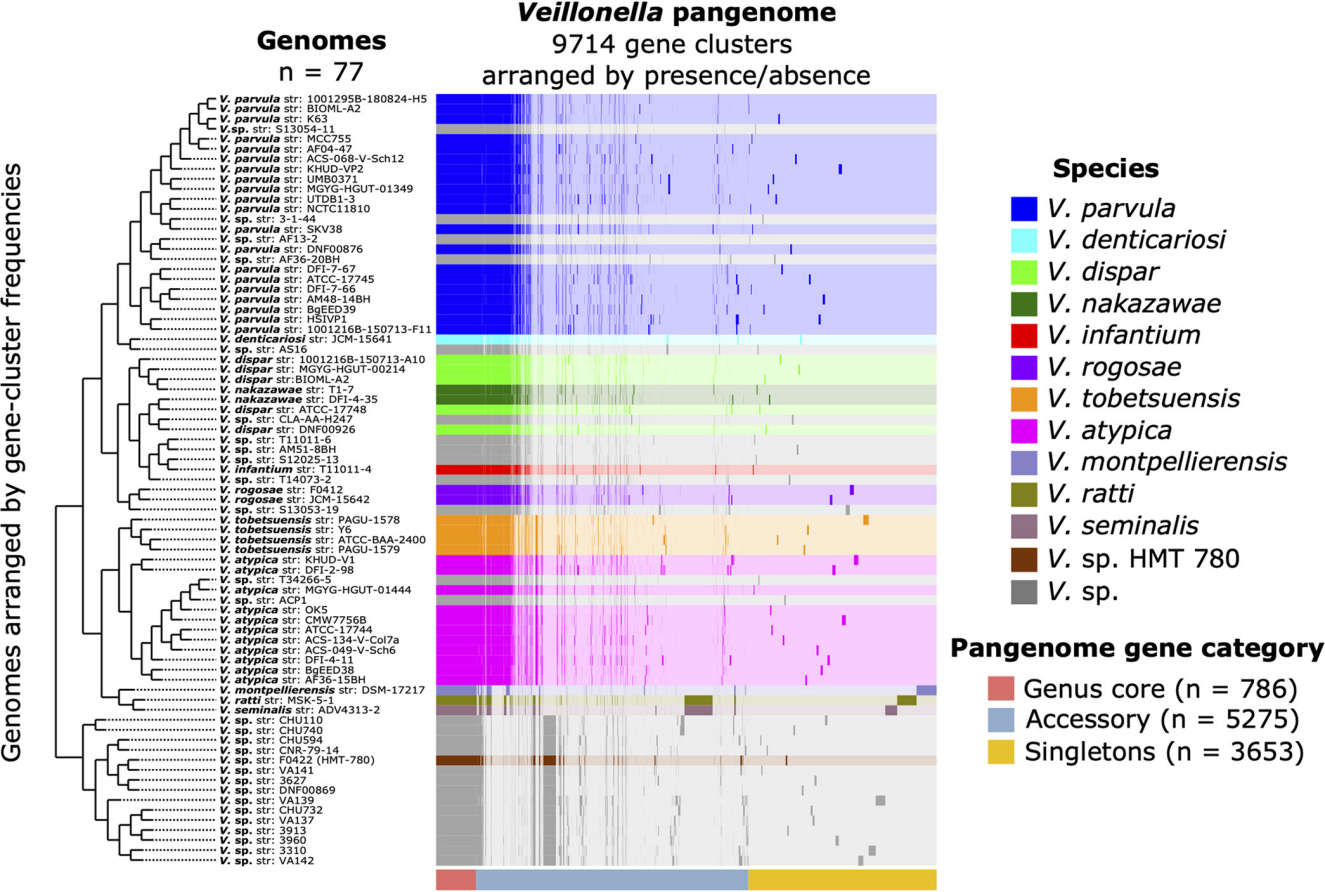
Human oral *Veillonella* genus pangenome constructed from available NCBI RefSeq genomes (*n* = 77). For all genomes, open reading frames (ORFs) were predicted, NCBI blastp was used to calculate amino acid sequence similarity between all possible gene pairs, and a Markov cluster algorithm was used to cluster similar sequences to identify homologous genes (i.e., gene clusters). The 9,714 distinct gene clusters of the pangenome include 786 core genes that occur in every genome, 3,653 singleton genes that occur in only a single genome, and 5,275 accessory genes that occur in more than one but not all genomes. Gene clusters are colored by species and arranged based on their presence or absence across the genomes. Genomes are hierarchically clustered based on gene cluster frequency (i.e., the number of representatives of each gene cluster present in each genome), which is shown by the dendrogram on the left. This pangenomic analysis results in distinct groups by species and predicts the species identities of the unnamed *Veillonella* genomes (*V*. sp.).

**FIG 2:**
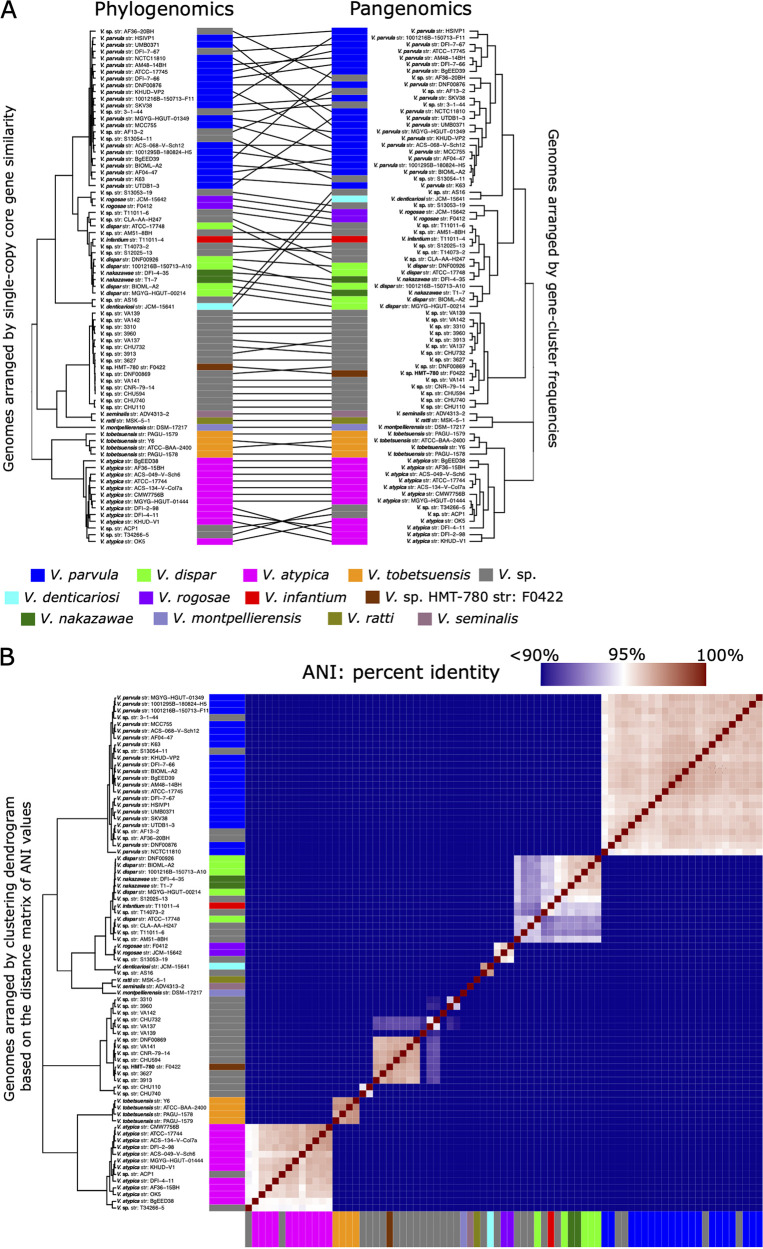
(A) Phylogenomic and pangenomic tree comparisons of *Veillonella* reference genomes cluster genomes into the same species-level groups. Rectangle color indicates species. The phylogenomic tree was constructed using maximum likelihood with 496 concatenated single-copy core genes. The pangenomic tree was constructed using the gene (*n* = 146,660) frequencies present in each genome. Lines connect rectangles that represent the same genome. (B) Average nucleotide identity (ANI) comparison of *Veillonella* reference genomes included in the pangenome. ANI represents the genome-level similarity at the nucleotide level between any two genomes and reveals distinct species, corroborating the results of the pangenome clustering. Rectangle color indicates species as in panel A. Color scale indicates genome percent similarity; 100% is red, 95% is white, and 90% and below is blue.

Pangenome construction identified distinct genomic groups within the *Veillonella* genus (Fig. 1). One clade included all named *Veillonella* species along with genomes identified in the National Center for Biotechnology Information (NCBI) database as *Veillonella* spp. that clustered within it, while a second clade consisted of the provisionally named *Veillonella* sp. Human Microbial Taxon 780 (HMT-780) along with *Veillonella* species genomes that clustered with it. The clade including named species clustered into three principal groups: group I, the Veillonella parvula group, consisted of *V. parvula*, V. denticariosi, and their cognate *Veillonella* species genomes; group II, the V. dispar group, consisted of *V. dispar*, V. infantium, V. rogosae, and their cognate *Veillonella* species genomes; and group III, the V. atypica group, contained the genomes of *V. atypica*, V. tobetsuensis, and the genomes identified in the NCBI database as *Veillonella* species that clustered with them. Genomes of V. montpellierensis, V. ratti, and V. seminalis, which were isolated from human nonoral sites (vagina, gut, and semen), did not cluster with any of the human oral genomes. We tested the congruence of these pangenomic groupings with phylogeny by generating a phylogenomic tree based on single-copy core gene clusters (SCG phylogeny). The SCG phylogeny matched well to the pangenome, although the organizations of genomes within certain clades were slightly different (Fig. 2A). One difference was the placement of *V. denticariosi*, and its single cognate *Veillonella* species genome, relative to the *V. parvula* and *V. dispar* groups. In the SCG phylogeny, *V. denticariosi* was an outgroup of the *V. parvula* clade, whereas in the pangenome, *V. denticariosi* was an outgroup of the *V. parvula* and *V. dispar* clades. We further assessed the taxonomic affiliations of *Veillonella* genomes by evaluating the average nucleotide identity (ANI) between all pairs of genomes. ANI is a similarity index between a given pair of genomes that can be used to identify genomes from the same species (26). A threshold of 95% ANI is often used to define bacterial species (27). The genomes of most species formed separate monophyletic clades within which all genomes shared >95% ANI, apart from a group containing *V. dispar*, V. nakazawae, *V. infantium*, and several genomes with no species designation in the NCBI database (Fig. 2B; see also Table S2 in the supplemental material). Within this group, application of a 95% ANI criterion would separate the type strain, *V. dispar* ATCC 17748, from the other 4 genomes named “*V. dispar*” in the NCBI database, while these 4 genomes share >95% identity with *V. nakazawae*. The taxonomy of this group may merit revision or reassignment. Overall, all three measures of genome relatedness (pangenomic gene content, phylogenomics, and ANI) agreed on the basic grouping of oral *Veillonella* genomes.

### Distribution of *Veillonella* genomes across human oral sites.

Mapping of metagenomic reads to the pangenome can reveal the relative distribution of species and individual genes across environments. We analyzed the distribution of *Veillonella* species across the oral cavity of healthy subjects by mapping metagenomic sequence data from the Human Microbiome Project (HMP) for individual sites onto our curated *Veillonella* reference genome set. In total, we mapped over 2,500,000,000 quality-filtered metagenomic short reads from 686 samples across nine oral sites (buccal mucosa [*n* = 183], supragingival plaque [*n* = 210], subgingival plaque [*n* = 19], dorsum of tongue [*n* = 220], hard palate [*n* = 1], palatine tonsil [*n* = 19], throat [*n* = 13], and saliva [*n* = 7]). Overall, based on the proportion of metagenomic reads that successfully mapped to *Veillonella* reference genomes, *Veillonella* organisms were abundant in the oral cavity, varying from 3% to 10% depending on site and accounting, on average, for 6.50% ± 0.72% (mean ± standard error [SE]) of the total metagenomic reads across all oral sites.

**FIG 3:**
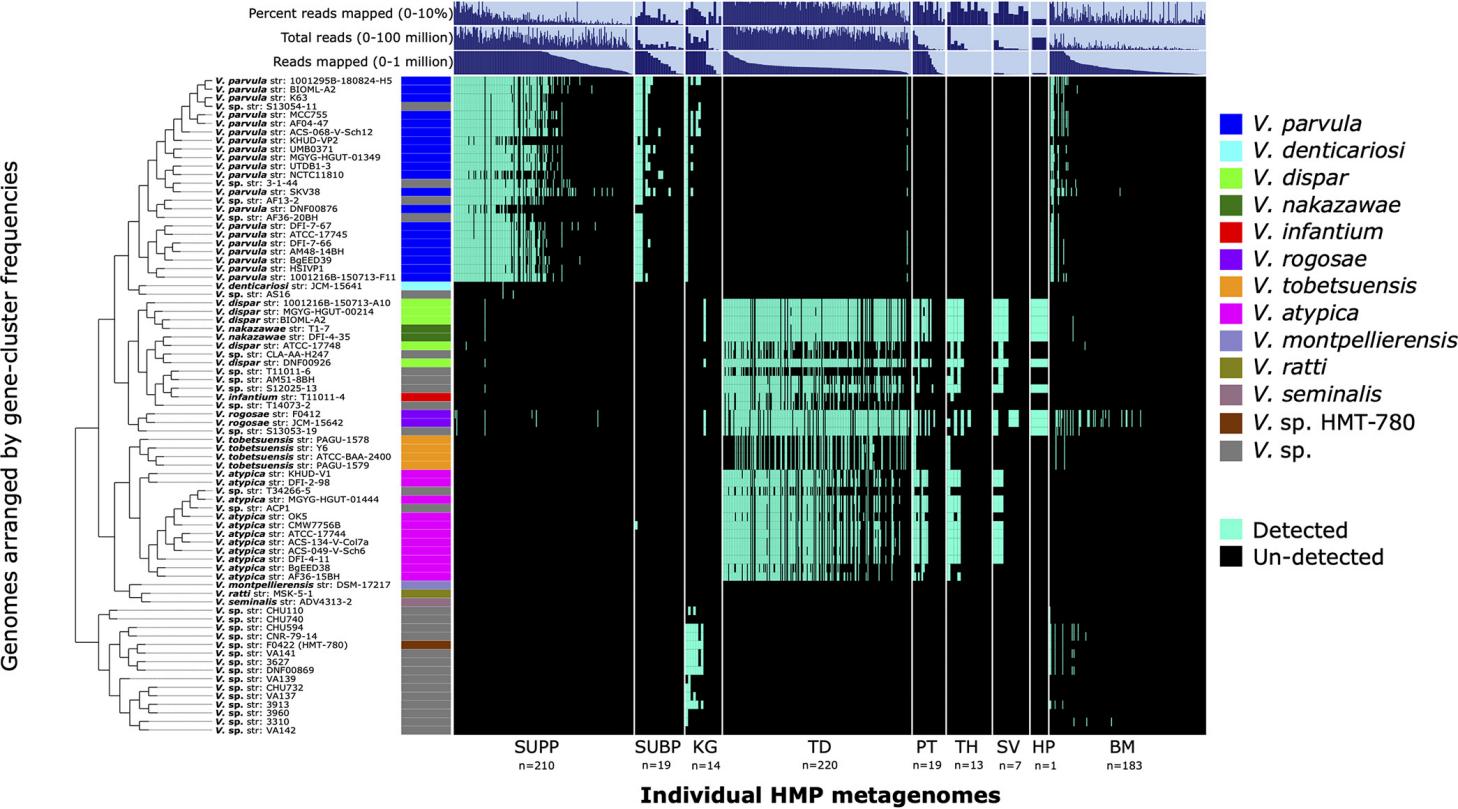
Detection plot of *Veillonella* species and strains in 686 Human Microbiome Project (HMP) metagenomic samples from 9 major oral sites. Each row displays the detection of a genome across all samples. A genome is detected (cyan) if at least 50% of its nucleotides have at least 1× coverage. If the genome is not detected in a sample, it is represented by a black bar. Samples are ordered by oral site and then by decreasing number of reads mapped to the set of genomes. From left to right, oral sites are supragingival plaque (SUPP), subgingival plaque (SUBP), keratinized gingiva (KG), (TD), palatine tonsil (PT), throat (TH), saliva (SV), hard palate (HP), and buccal mucosa (BM). Additional data are shown for total mapped reads, total sample reads, and percentage of reads mapped, which may be taken as a measure of genus abundance per metagenomic sample.

We classified genomes as detected if there was at least 1× depth of coverage for at least 50% of nucleotides for a given genome. Based on this detection metric, we found that human and oral *Veillonella* genomic groups are site specialists (Fig. 3). Reads from supragingival and subgingival plaque samples mapped almost exclusively to genomes of the *V. parvula* group, whereas reads from tongue dorsum, palatine tonsil, throat, and saliva samples mapped to the *V. atypica* and *V. dispar* groups. Genomes in the *Veillonella* sp. HMT-780 group were not detected in reads from supra- and subgingival plaque or tongue dorsum, but did recruit reads from keratinized gingiva and some buccal mucosa samples. Reads in some keratinized gingiva and buccal mucosa samples also mapped to *V. parvula*. The *V. denticariosi* genome, along with its cognate *Veillonella* species genome, was detected in only two supragingival plaque samples. The nonoral *V. montpellierensis*, *V. ratti*, and *V. seminalis* negative-control genomes were not detected in any metagenomic samples, thus supporting the integrity of our mapping results. Mapping results were independent of donor gender.

**FIG 4:**
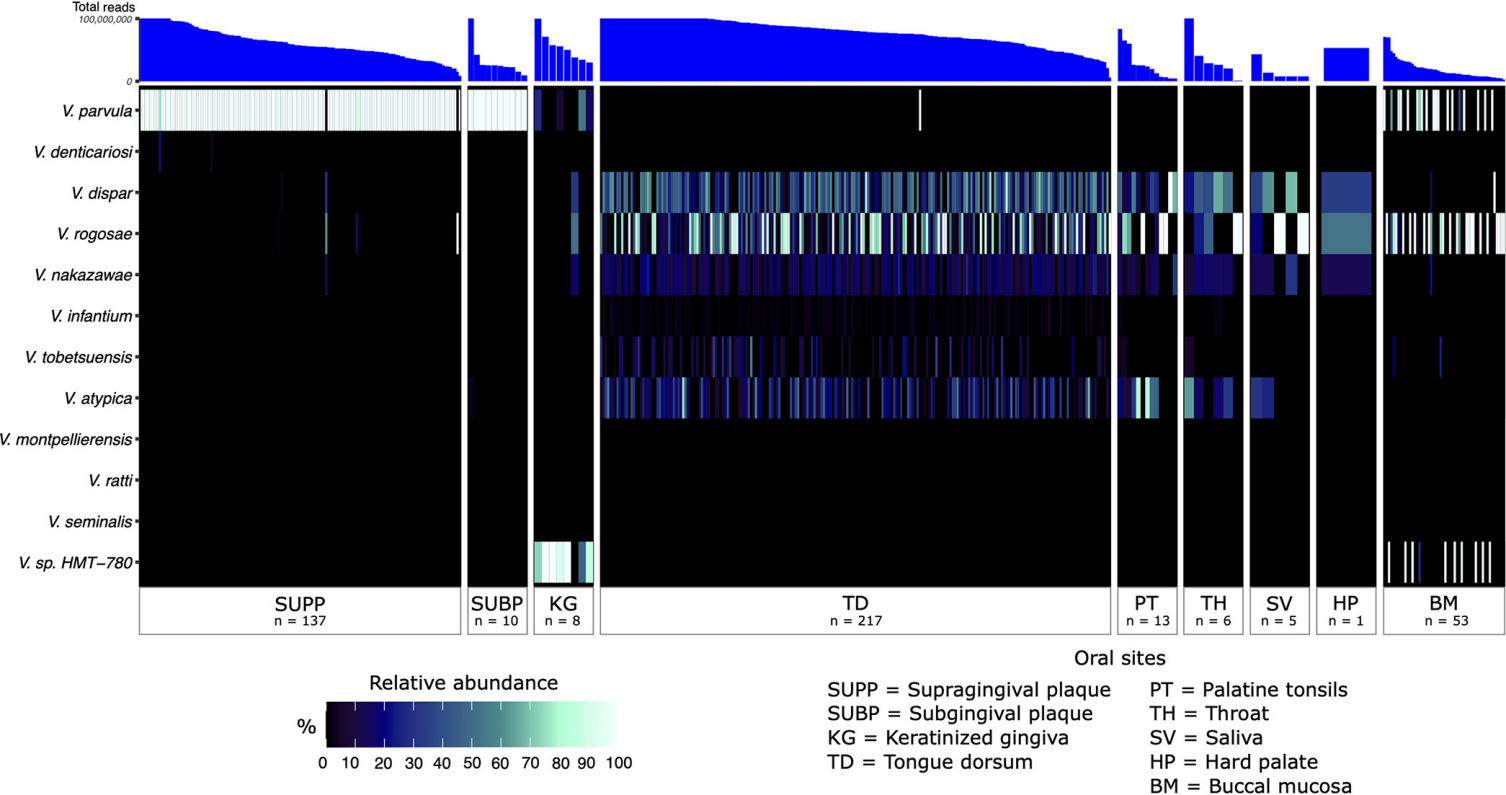
Heat map showing the relative abundances of *Veillonella* species with respect to the set of reference genomes across 9 major oral sites: supragingival plaque, subgingival plaque, keratinized gingiva, tongue dorsum, palatine tonsil, throat, saliva, hard palate, and buccal mucosa. The relative abundance for each species represents the sum of relative abundances of each reference genome from that species, calculated from the mean depth of coverage across nucleotide positions in the 2nd and 3rd quartiles (the interquartile range) after nucleotides were ranked by their depth of coverage and then divided by the sum of mean coverages of all genomes within a metagenomic sample. The rows and columns correspond to individual species and Human Microbiome Project metagenomic samples, respectively. Only metagenomes in which at least one reference genome was detected (i.e., at least 50% of its nucleotides have at least 1× coverage) were included. The number of metagenomes for each oral site in which a species was detected is listed. For clearer visualization, we inflated the width of oral sites with small sample sizes. Metagenomes are in descending order for each oral site by the total number of quality filtered reads.

Complementary to detection is abundance, which can reveal potentially large differences in the number of reads recruited (i.e., depth of coverage). For each metagenome, we analyzed the relative abundance of each *Veillonella* genome by evaluating the mean depth of coverage of each detected *Veillonella* genome relative to the total depth of coverage of all detected *Veillonella* genomes. Summing this abundance metric across all genomes in a species group revealed the species that are dominant in each oral habitat. Generally, the distribution patterns of *Veillonella* species in the healthy human oral cavity as measured by this abundance metric are similar to those of the detection metric (Fig. 4). Taken together, our results reveal that *Veillonella* species are specialized to ecologically related sites: *V. parvula* to both supra- and subgingival plaque; *V. dispar*, *V. atypica*, *V. rogosae*, *V. tobetsuensis*, and *V. nakazawae* to tongue dorsum, throat, and tonsils; and *Veillonella* sp. HMT-780 to keratinized gingiva.

Competitive read mapping provides limited information about the correspondence between the reference genomes and the strain-level composition of the metagenome. Reads whose best matches are equidistant to multiple reference genomes will be distributed at random among these genomes; therefore, the fact that multiple reference genomes from a single species pass the detection threshold does not necessarily signify that multiple strains of that species are present in the metagenome. However, differences in genome-level mapping can indicate which sequenced genomes are most representative of the *Veillonella* populations in the samples. The mapping results for each reference genome indicated that not all strains of a given species were equally detected in the oral cavity (Fig. 3). Some *V. parvula*, *V. dispar*, and *V. atypica* reference genomes recruited fewer reads than others, reflecting their poor resemblance to the populations of these three species present in the samples. Overall, genomes within each clade showed similar distribution patterns across oral sites, indicating a lack of subspecialization to different oral sites by strains within a species.

**FIG 5:**
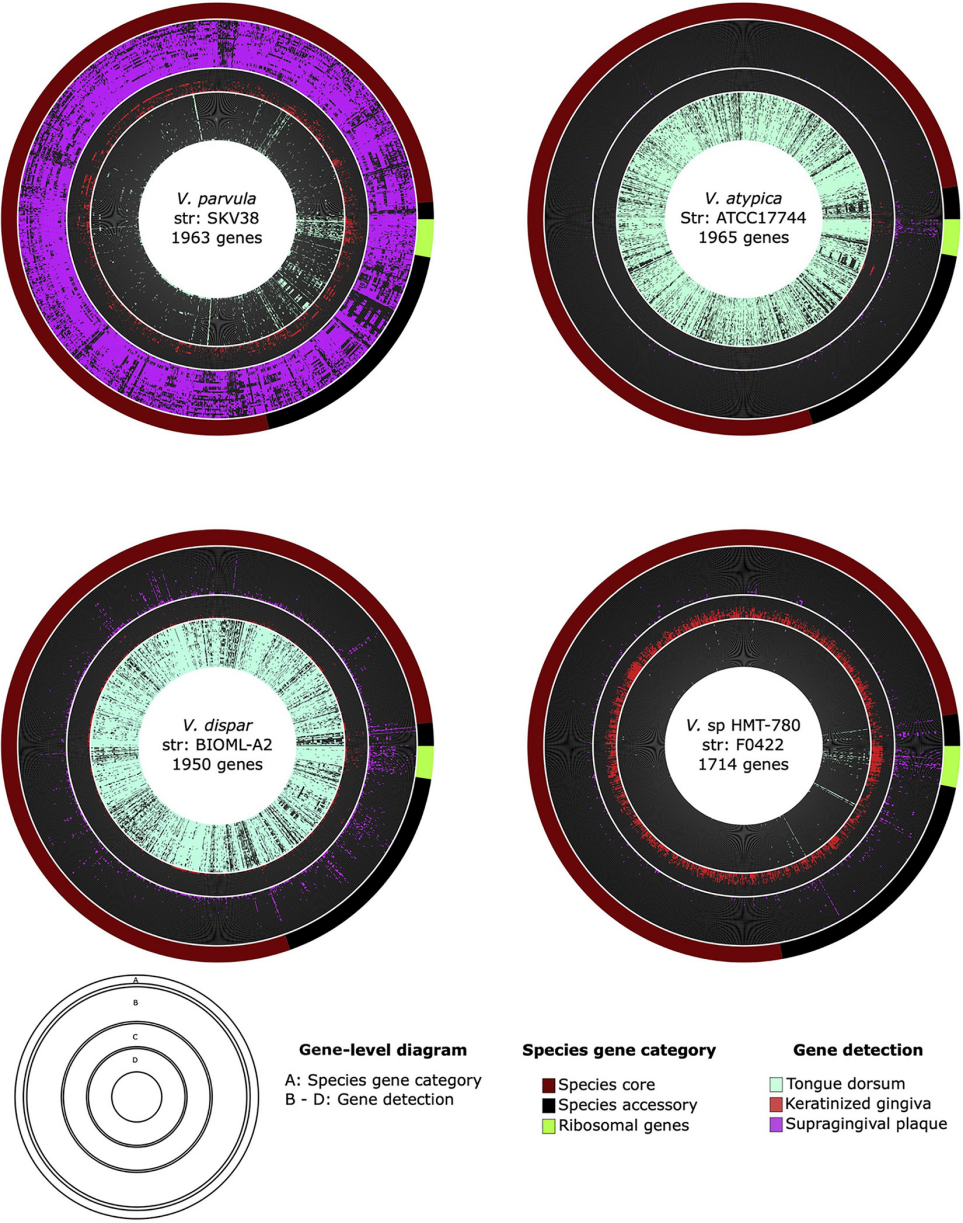
Gene-level detection diagrams for select *Veillonella* genomes that showed clear patterns of site specialization. Here, we display radial gene-level detection maps for four genomes showing the detection of genes across a subset of the top 30 metagenomic samples ranked by median coverage from 2 oral sites (supragingival plaque and tongue dorsum) and all available 14 metagenomic samples from keratinized gingiva (red). We included *V. parvula* strain SKV38, *V. dispar* strain BIOML-A2, *V. atypica* strain ATCC 17744, and *Veillonella* sp. HMT-780 strain F0422 to represent species that were the most abundant based on mean coverage within their respective preferred oral site. A gene was classified as detected within an oral site when at least 90% of the nucleotides of the gene had at least 1× coverage. Genes are ordered according to the gene category for the respective species as indicated in the outermost layer for each diagram. A group of ribosomal genes translated using hidden Markov models via the Anvi’o program *anvi-run-hmms* are indicated in bright green.

Inspection of gene-level coverage can be used to assess whether coverage is distributed evenly across a genome, as would be expected if the presence and absence of genes in the reference genome is an accurate representative of genomes in the mouth. Having found clear patterns of species site specialization using our genome detection and relative-abundance metrics, we then further examined coverage at a gene-level scale. We classified a gene as detected if at least 90% of its nucleotides had at least 1× coverage; this 90% threshold is stringent yet allows for hypervariable regions that may differ between reference genomes and environmental genomes. Fig. 5 shows radial gene-level detection maps of four genomes (*V. parvula* SKV38, *V. dispar* BIOML-A2, *V. atypica* ATCC 17744, and *Veillonella* sp. HMT-780) that had high relative abundance within their preferred sites (supragingival plaque, tongue dorsum, tongue dorsum, and keratinized gingiva, respectively). These gene detection maps show that for each genome, a large proportion of genes were detected in only one of the three oral sites. Three genomes, those of *V. parvula* SKV38, *V. dispar* BIOML-A2, and *V. atypica* ATCC 17744, showed preference for oral sites for which many metagenomic samples with high numbers of reads were available. For the 30 metagenomic samples with greatest median coverage as shown in Fig. 5, gene detection was clearly distributed evenly across the genome, with 78% ± 7% (mean ± 1 standard deviation) of genes detected on average within the preferred oral site. One genome, that of *Veillonella* sp. HMT-780, attracted reads specifically from keratinized gingival samples. For this site, only 14 metagenomic samples were available, and most of them had few total reads compared to samples from other oral sites, limiting the detection sensitivity (Fig. 3). Consequently, the proportion of genes detected for *Veillonella* sp. HMT-780 was lower and their evenness across the genome was less evident. However, like for the other genomes, gene-level detection was site specific. Also apparent in the gene detection maps is that a small proportion of genes attracted reads from multiple habitats. Many of these genes were annotated with highly conserved functions (e.g., ribosomal genes, cell division, and DNA replication) or transposable elements and thus may represent cross-mapping to different taxa that share high similarity in these genes or share elements that have been horizontally acquired. In sum, gene-level coverage analysis confirmed the site tropisms suggested by the whole-genome-level coverage analysis and extended that analysis by identifying individual genes present in specific oral sites.

### Functional analysis of detected *Veillonella* genomes across human oral sites.

**FIG 6:**
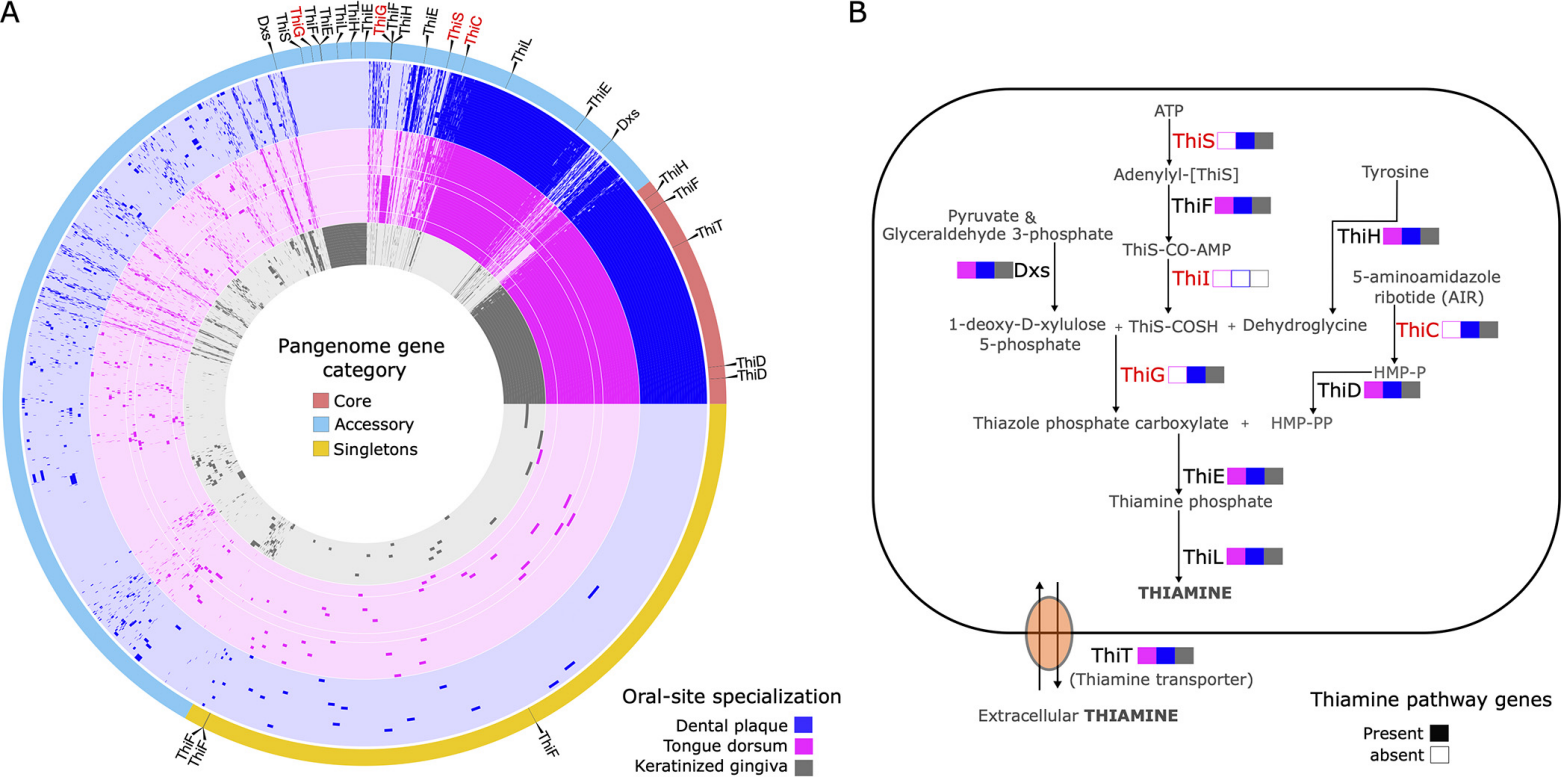
(A) A pangenome of human oral *Veillonella* reference genomes (*n* = 72) was constructed for supragingival plaque, keratinized gingiva, and tongue dorsum genomes. Species and strains not detected in these oral sites were excluded. Gene cluster arrangement, genome color, and genome arrangement are like the previously constructed pangenome (Fig. 1). Thiamine pathway-associated genes are indicated with a black line in the pangenome gene category outer layer and pointed out by a black triangle. (B) The diagram illustrates the enzymes, metabolites, and transporters involved in the thiamine pathway in human oral *Veillonella* species. The presence of a gene is indicated with a filled color box; empty boxes indicate that no gene was found in any genome assigned to the respective oral site. The colors designate oral sites, and genomes were grouped into an oral site based on detection and relative abundance patterns (Fig. 3 and 4).

Metapangenomics can be used to reveal genetic variation that drives community structure of the oral microbiome by identifying gene functions that are core to a group of genomes at a specific oral site and absent from the genomes of other sites. Based on their detection and abundance profiles (Fig. 3 and Fig. S1), we grouped *Veillonella* genomes according to their site preferences. Three metabolic modules were significantly enriched in supragingival plaque and keratinized gingiva genomes compared to tongue dorsum genomes based on an adjusted *q* value threshold of 0.01 (Table S3). This included two modules associated with the thiamine biosynthesis pathway (vitamin B_1_; KEGG module identifiers [ID] M00896 and M00127) and the reductive pentose phosphate cycle (KEGG module ID M00167), which were consistently incomplete in tongue dorsum genomes (Table S3). Closer inspection of the presence and absence of individual genes that make up the thiamine biosynthesis pathway revealed that the genomes of *V. dispar*, *V. atypica*, *V. tobetsuensis*, and *V. rogosae*, all of which appear to be tongue dorsum specialists, are missing genes that play an important role in the thiazole and hydroxymethylpyrimidine phosphate moiety complexes required to produce thiamine *de novo* (Fig. 6). Additionally, both dental plaque and tongue dorsum specialists had a complete thiamine salvage pathway (KEGG module ID M00899), which was missing entirely from keratinized gingiva specialists. In a similar light, closer inspection of module completeness for the reductive pentose phosphate cycle revealed that *V. atypica* and *V. tobetsuensis* genomes are missing fructose-1,6-bisphosphatase II (GlpX), which is present in the *V. parvula* and *V. dispar* genomes (Table S3).

Keratinized gingiva specialists (i.e., *Veillonella* sp. HMT-780) were distinguished from both dental plaque and tongue dorsum genomes by the enrichment of three metabolic pathways associated with carbohydrate metabolism, including the oxidative-phase pentose phosphate pathway (M00006), which converts glucose-6-phosphate to ribulose-5-phosphate, and two pathways associated with galactose metabolism: the galactose degradation Leloir pathway (M00632) and a nucleotide sugar biosynthesis (NSB) pathway that converts galactose to UDP-galactose (M00554). The Leloir pathway enzymes include aldose 1-epimerase (GalM), galactokinase (GalK), galactose-1-phosphate uridylyltransferase (GalT), and UDP-galactose 4-epimerase (GalE), which are responsible for the conversion of galactose to glucose-1-phosphate. The NSB pathway enzymes include a subset of the Leloir pathway: GalT and GalE. Closer inspection of all functional annotations in our data set (i.e., NCBI clusters of orthologous genes [COGs], Pfam, and KEGG) revealed that *Veillonella* dental plaque and tongue dorsum specialists lack GalM and GalK but have GalT and GalE. All *Veillonella* genomes in our data set have the genes required to metabolize fructose via the Embden-Meyerhof-Parnas (EMP) pathway, as well as a complete tagatose-6-phosphate pathway, in which d-galactose or lactose is converted to d-galactose-6-phosphate and further metabolized through tagatose derivatives (28).

In addition to assessing the completeness of metabolic pathways, we analyzed functional associations derived from the NCBI COG20, Pfam, and KEGG databases to identify gene functions that were significantly enriched in certain *Veillonella* groups (Table S4). Noteworthy enriched gene functions were as follows: eight functions were significantly enriched in both supragingival plaque and keratinized gingiva compared to tongue dorsum genomes, the top three of which included thiamine biosynthesis genes: the sulfur carrier protein (ThiS), thiazole synthase (ThiG), and 4-amino-2-methyl-5-hydroxymethylpyrimidine synthase (ThiC). Only a single function, magnetosome formation protein MamQ, was enriched in both tongue dorsum and keratinized gingiva compared to supragingival plaque genomes. Magnetosomes are organelles comprising magnetic mineral crystals surrounded by a phospholipid bilayer that are thought to play an important role in cell movement to optimize growth and survival (29). Thirty-two functions were significantly enriched in both dental plaque and tongue compared to keratinized gingiva genomes, including beta-lactamase (LemA), which is a protein that confers resistance to antibiotics, namely, penicillins (30). Adhesins play an important role in biofilm formation and may be a factor contributing to *Veillonella* species-level niche partitioning in the oral microbiome (31). However, we did not find evidence of differential enrichment of any genes directly associated with adherence, such as adhesins, between the genomes of *Veillonella* site specialists.

The pangenome was constructed using a set of isolate reference genomes that were dereplicated based on a 98% ANI threshold, which may result in the loss of information on variability in the accessory gene content among representatives from the same species. To evaluate the potential cost of removing genomes, we constructed additional pangenomes using genome sets dereplicated at 99% and 99.5% ANI. Raising the ANI threshold increased the size of the pangenome by 2 and 5 genomes, respectively (Fig. S1 and S2). Increasing the ANI threshold also modestly decreased the number of core and singleton gene clusters and increased the number of gene clusters categorized as accessory (Fig. S3). Inspection of the singleton gene clusters shows that many of the additional protein sequences are functionally associated with mobile elements or annotated as hypothetical proteins (Table S5). One genome (*V. dispar* strain BIOML-A3) that was added to the 99.5% ANI threshold pangenome contained a relatively large number of singleton gene clusters, for many of which the top blast hit was a protein sequence from the bacterial genus *Eggerthella* and likely reflects bacterial contamination in the genome. We did not find any evidence of additional genes associated with vitamin biosynthesis or carbohydrate metabolism that would alter our previous results. We thus conclude that the 98% ANI threshold for genome dereplication did not adversely affect the results of our analysis.

## DISCUSSION

Metapangenomics can provide information on ecological distribution and genetic variation among oral bacterial species and strains. Using this approach, we found that human oral *Veillonella* species demonstrated strong patterns of site specialization, such that individual species were most abundant in either dental plaque, keratinized gingiva, or the tongue dorsum, throat, and tonsils. Analysis of unique or overrepresented genes and functions among site-specific genome groups identified genes associated with carbohydrate metabolism and two key metabolic pathways, including thiamine biosynthesis and the pentose phosphate cycle. These genes associated with adaptation to an oral site may enable different *Veillonella* species to occupy different habitats and thus help explain their oral site tropisms.

Our approach takes advantage of a cornucopia of publicly available data to systematically analyze the genomic content of *Veillonella* species in the oral microbiome within an ecological framework of niche partitioning across oral habitats. The combination of pangenomic analysis using genome sequences from cultivars and oral metagenomic information provided unique species-, strain-, and gene-level insights. First, because marker genes, such as the 16S rRNA gene, fail to distinguish some closely related *Veillonella* species (16, 17), metapangenomics improved identification of species based on genomic coherence and enabled identification of strains for *in vitro* model systems that more accurately represent the oral community. Second, our approach goes beyond characterization of a species’ site tropism and analyzes gene distribution and abundance across genomes and in samples from the mouth. The relevance of cultivars to most of the organisms living in the mouth is generally not known, and successful mapping depends on the set of reference genomes being sufficiently similar at the nucleotide level to environmental genomes. Nonetheless, we obtained extensive mapping, resulting in relative abundance estimates similar to those obtained by 16S rRNA gene sequencing (7). This validates the overall relevance of cultivars, but follow-up mechanistic studies using experimental manipulations are necessary to validate our findings.

Although pangenomic analysis is typically performed at the species level, analysis at the genus level provides several benefits. First, the clustering of genomes identified in databases only to the genus level (i.e., *Veillonella*) with genomes identified to the species level greatly augments the number of genomes in the pangenome analysis, thus enabling a greater representation of species and strain genetic variation. This point is most dramatically exemplified for the *Veillonella* sp. HMT-780, for which the single genome available in the NCBI database was augmented by 14 additional genomes that clustered with it. Other oral species were also augmented: *V. atypica* by 2 genomes, *V. parvula* by 4 genomes, *V. rogosae* by 2 genomes, and *V. dispar* by 5 genomes. Analysis of other oral genera by pangenomics has resulted in similar findings (32). Second, relationships among the species comprising the genus can be readily visualized. For example, the difference in gene content between the *Veillonella* sp. HMT-780 group and the other *Veillonella* oral genomes is much greater than the differences between any of the other oral genomes, indicating that human oral *Veillonella* can be divided into two major clades, one defined by *Veillonella* sp. HMT-780 and the other containing the *V. parvula*, *V. dispar*, and *V. atypica* groups. The last three groups share a large set of core genes and are distinguished from each other by small sets of unique species core genes. Third, possible species misclassifications become apparent. For example, our results revealed that the *V. dispar* and *V. nakazawae* genomes were similar in gene content and had nearly identical distribution patterns in the human oral cavity. *V. nakazawae* was originally classified as a novel species by comparing two strains of previously unknown Gram-negative cocci, T1-7^T^ and S6-16, to a single *V. dispar* type strain, ATCC 17748, in terms of catalase production, partial *dnaK*, *rpoB*, and *gltA* sequences, average ANI, and digital DNA-DNA hybridization (dDDH) values (33). However, our analysis suggests that *V. nakazawae* should be considered a later synonym of *V. dispar*. In addition to *V. nakazawae*, our analysis included genomes for two species not listed in eHOMD (http://www.homd.org): *V. tobetsuensis* and *V. infantium*. Our results from both phylogenomics and ANI indicate that *V. tobetsuensis* is a sister group to *V. atypica*, whereas *V. infantium* clusters within *V. dispar* and, like *V. nakazawae*, should be considered a later heterotypic synonym of *V. dispar* unless the *V. dispar* cluster is subdivided into distinct species.

Comparative genomic analysis can be used to reveal the potential molecular underpinnings that drive *Veillonella* site specialization in the oral microbiome. One important factor in determining species colonization is the availability of macronutrients, such as carbohydrates, fats, and proteins. *Veillonella* organisms are known to rely on by-products of other heterotrophic bacteria, primarily pyruvate and lactate, as a source of energy (34–36) and are thought to be unable to use carbohydrates or amino acids as an energy source (3, 4). Our findings suggest that *Veillonella* species that inhabit different oral sites metabolize lactate and carbohydrates in slightly different ways. First, the gene for lactate racemase (LarA), which enables the consumption of both l- and d-enantiomers of lactate in highly anoxic environments (37, 38), was enriched in tongue dorsum specialists compared to supragingival plaque and keratinized gingiva specialists. Lactate is mostly found in its natural l-form, whereas d-lactate is a by-product of some fermentation processes (39) and lactic acid-producing bacteria (LAB) such as streptococci and lactobacilli (40). Thus, LarA may enhance the ability of *Veillonella* tongue dorsum specialists to utilize localized pockets of high concentrations of d-lactate produced by LAB on the tongue. However, the genes for both l-lactate dehydrogenase (LdhL) and d-lactate dehydrogenase (LdhD), which convert l-lactate and d-lactate to pyruvate and back, respectively, were found in all oral *Veillonella* species in our data set regardless of site preference. Consequently, LarA may act as a rescue enzyme to ensure d-lactate metabolism under physiological conditions where LdhD production is not sufficient. Second, in anaerobic environments C_5_ sugars are released as by-products of aerobic bacteria and subsequently taken up by anaerobic bacteria (41). In this study, we found that *Veillonella* tongue specialists are further differentiated from dental plaque specialists by having an incomplete pathway for the reductive pentose phosphate cycle, although the pathway is lacking only in *V. atypica* and *V. tobetsuensis*, a subset of tongue specialists.

We found that the tagatose-6-phosphate pathway (i.e., Lac genes) is conserved in all oral *Veillonella* species. In this pathway, galactose metabolism occurs by transforming galactose-6-phosphate to glycolysis intermediates via a series of enzymes: galactose-6-phosphate isomerase, tagatose-6-phosphate kinase, and tagatose diphosphate aldolase. A complete tagatose-6-phosphate pathway suggests that oral *Veillonella* could utilize galactose as a source of energy in addition to lactate. Additionally, *Veillonella* keratinized gingiva specialists (HMT-780 group) also have a complete Leloir pathway (i.e., Gal genes) not found in *Veillonella* tongue dorsum and dental plaque specialists. This suggests that *Veillonella* HMT-780 has an enhanced capability to metabolize galactose compared to other oral *Veillonella* species. Our results support a previously published comparative pangenomic analysis of eight human oral *Veillonella* species that used one genome for each species and found that oral *Veillonella* organisms have conserved pathways that utilize carbohydrates other than lactate as an energy source, namely, fructose and tagatose (13). Expanding on this finding, here we propose that *Veillonella* HMT-780 and other members of the *Veillonella* genus may have evolved to utilize galactose as an additional carbon source.

Another important factor determining species colonization is the availability of micronutrients, such as inorganic cofactors (e.g., zinc ions) and coenzymes that maintain normal metabolism. Our findings indicate that *Veillonella* tongue specialists lack the ability to synthesize the vitamin thiamine *de novo*. Thiamine plays a major role as a coenzyme in many essential metabolic pathways, including glycolysis, the pentose phosphate cycle, and the citric acid cycle (42). Bacteria can either synthesize thiamine *de novo* or acquire precursors or mature thiamine via transport from their environment. Thiamine synthesis requires the independent production of two major precursors, including the thiazole and pyrimidine moieties (42). To produce thiazole, a sulfur carrier protein (ThiS) provides an initial source of a sulfur group, which is combined with dehydroglycine and deoxy-d-xylulose-phosphate (DXP) with the help of thiazole synthase (ThiG). Tongue dorsum *Veillonella* organisms lack ThiS and ThiG and are thus unable to synthesize the thiazole moiety *de novo*, whereas dental plaque and keratinized gingiva specialist *Veillonella* spp. possess the full pathway.

The evolutionary loss of costly genes required to produce thiamine is expected where an organism could obtain thiamine or its precursors through other less costly means (43). Concentrations of free thiamine in the human body are thought to be exceptionally low due to tight sequestration of the vitamin, efficient recycling (44), and the rapid degradation brought on by physical and chemical factors such as UV radiation, temperature, and pH (45, 46). Thus, for thiamine synthesis to be lost, a reliable source of thiamine must be present in the immediate environment of the microbe. The distance over which a nutrient can be provided depends on its diffusion constant and the rate at which it is taken up by intervening cells, but it is generally less than 20 μmol when uptake rates are high (47). Therefore, we hypothesize that the tongue dorsum environment allows *Veillonella* to acquire thiamine or the thiazole precursor from other bacteria in its immediate neighborhood, whereas the dental plaque and keratinized gingiva environments do not.

The complexity of the microbial community of the oral cavity suggests that interactions with other bacterial taxa may be an important determinant of *Veillonella* colonization patterns. *Veillonella* species may compete with other bacteria for nutrients or may form commensal or mutualistic interactions that stimulate growth and survival of one or more interactors. With respect to thiamine, a recent systematic genome assessment of B-vitamin biosynthesis found that approximately one-third of oral bacterial species are capable of *de novo* thiamine synthesis (48), indicating that different taxa may encode different portions of the thiamine pathway. Thus, *Veillonella* tongue specialists may acquire thiamine or precursor chemicals from neighboring bacteria. Indeed, the thiamine transporter ThiT (49) is present in all oral *Veillonella* genomes analyzed in this study. A recent spatial analysis using fluorescence *in situ* hybridization has shown that the taxon Rothia mucilaginosa, which is capable of *de novo* thiamine synthesis, is present in tongue dorsum bacterial consortia in high abundance (50). This finding suggests that *Veillonella* tongue specialists may acquire thiamine from nearby *R. mucilaginosa*. Conversely, *Veillonella* dental plaque specialists may have developed the ability to synthesize thiamine *de novo* to cope with a lack of freely available thiamine or its precursors in the environment. Indeed, it appears that dominant dental plaque taxa, including Streptococcus, *Actinomyces*, and Rothia dentocariosa (7), lack the ability to produce thiamine *de novo* (48), although in *Corynebacterium*, which has been described as a base of microbial community structure and interactions in dental plaque (7), thiamine synthesis appears to be conserved (48). These studies highlight the potential for vitamins to play a critical role in driving microbiome dynamics and ultimately the distribution of *Veillonella* in the human oral cavity.

In summary, our systematic metapangenomics approach has established the oral site-specific tropisms of *Veillonella* species, has provided insight into the genetic determinants of the ecology of the genus, and has illuminated the potential role of vitamins and carbohydrates in determining *Veillonella* distribution patterns and interspecies interactions. Spatial niche partitioning has been discovered for multiple taxa within the oral microbiome (8, 32, 51), as well as different microbial species across a large diversity of habitats, such as riverine floodplains, soil, and plastic debris in the ocean (52–54). Our results indicate a need for further research into whether the presence or absence of pathways for synthesis of certain nutrients is a determining factor in defining the niche space of a bacterium or represents evolutionary change resulting from life in the niche. The oral microbiome, with its diverse range of bacterial taxa living on a variety of distinct habitats, such as teeth, tongue, cheeks, and gums, which differ in chemistry, topography, and stability, provides a useful system for testing ecological theories about the causes and consequences of niche partitioning.

## MATERIALS AND METHODS

The following analyses were conducted primarily using the Anvi’o v7 platform (55) with Python v3.7.9.

### *Veillonella* reference genomes.

To assess whether *Veillonella* species are site specialists or generalists in the human oral cavity, we first developed a reference genome set representative of natural *Veillonella* populations in the human oral microbiome. We obtained publicly available sequenced genomes of *Veillonella* species from the National Center for Biotechnology Information (NCBI) database (all genomes downloaded 15 December 2021) and selected genomes that were of sufficient quality to be designated reference sequences (RefSeq). Of 113 genomes meeting this criterion, 11 were excluded because they were isolated from a nonhuman host species and 2 were excluded because they were duplicate strains. Of the resulting 100 genomes, 49 had species names that matched species contained in eHOMD (http://www.homd.org), including *Veillonella* sp. HMT-780; 20 genomes belonged to species not listed in eHOMD and 31 genomes were identified in NCBI only to the genus level, i.e., *Veillonella*. Information about genome genus, species, strain, BioSample, BioProject, isolation host, isolation site, RefSeq status, type strain, disease association, and submitter can be found in Table S1.

We then further filtered the set of reference genomes so that each genome included in the set shared no more than 98% average nucleotide identity (ANI) with any other genome, as well as had a completeness of ≥90% and a contamination level of <5% estimated by CheckM (56). ANI between genomes was estimated using the Anvi’o program *anvi-compute-genome-similarity* with the program pyANI (v0.2.10) and ANIblastall, which used the blastn method (57). The result was 46 high-quality reference genomes from 8 species that represent the diversity of *Veillonella* within the human oral microbiome, as well as 28 genomes identified only to the genus level. In addition, we included 3 high -quality genomes that represent *Veillonella* species isolated from human hosts but not known to be present in the human oral cavity, including *V. montpellierensis*, *V. ratti*, and *V. seminalis*. At the time of this study, the taxonomy check for the *V. ratti* genome was identified in the NCBI database as inconclusive and the genome may actually be a *V. seminalis* genome (58). These nonoral genomes function as a negative control for downstream analyses. The total number of genomes used to construct a genus-level *Veillonella* pangenome was 77.

### Constructing an oral *Veillonella* pangenome.

Pangenome construction was adapted from previously developed methods (20, 32) and carried out using the Anvi’o platform. First, all contigs within each reference genome that were <300 nucleotides (nt) were removed and noncanonical nucleotide letters were replaced with the letter “N.” Each genome was then converted into an Anvi’o-compatible contig database using *anvi-gen-contigs-db*. Open reading frames (ORFs; here also referred to as genes) were identified for each genome using Prodigal (v2.6.3) (59). Functional annotation of ORFs was done using the scripts *anvi-run-hmms* to find bacterial single-copy genes (Bacteria71 SCG set) (60, 61) with hidden Markov model (HMM) profiles, *anvi-run-ncbi-cogs* using blastp (v2.10.1+) to annotate with the cluster of orthologous genes (COGs) database (version COG20) (62), and *anvi-run-pfams* and *anvi-run-kegg-kofams* with hmmscan from HMMER (v3.3.1) to functionally annotate with Pfams (v34.0) (63) and KOfams/KEGG modules (v97.0) (64), respectively. To construct the pangenome, blastp was used to compute amino acid identity between all possible ORF pairs, and weak matches were removed by employing the –minbit criterion of 0.5. A Markov cluster algorithm (MCL) was then used to group ORFs into putatively homologous groups termed gene clusters (mcl-inflation = 10), and amino acid sequences within gene clusters were aligned with MUSCLE (v3.8.1551) (65). Hierarchical clustering was performed across gene clusters and genomes using Euclidean distance and Ward linkage.

### *Veillonella* phylogeny.

To assess how well the pangenome organized the *Veillonella* reference genomes, we constructed a phylogenetic tree based on the concatenated amino acid sequences of single-copy core genes present in all *Veillonella* reference genomes. To construct the phylogenetic tree, we selected informative single-copy core gene clusters from the pangenome using *anvi-get-sequences-for-gene-clusters* (parameters: --min-num-genomes-gene-cluster-occurs 77 --max-num-genes-from-each-genome 1 --concatenate-gene-clusters), which resulted in 496 single-copy gene clusters that contained a total of 38,192 genes. Genes were concatenated into a single fasta file, trimAl (v1.2) (66) was used to remove all columns with gaps in more than 50% of the sequences, and IQ-tree (v1.6.12) (67) was used to build a phylogenetic tree using maximum likelihood and the Whelan and Goldman (WAG) method with 1,000 bootstrap replicates.

### Distribution of *Veillonella* genomes across human oral sites.

The metagenomes used in this study were obtained from the Human Microbiome Project portal (https://portal.hmpdacc.org/) using the following search parameters: oral sites (buccal mucosa [*n* = 183], supragingival plaque [*n* = 210], subgingival plaque [*n* = 19], dorsum of tongue [*n* = 220], hard palate [*n* = 1], palatine tonsil [*n* = 19], throat [*n* = 13], and saliva [*n* = 7]), Healthy Human Study (HHS), fastq files (FASTQ), and whole-genome sequencing (wgs_raw_seq_set). Metagenomes consisted of ~100-bp paired-end reads sequenced from samples collected from nine oral sites in phases I and II of the Human Microbiome Project. Quality filtering was performed using *iu-filter-quality-minoche* (68), which keeps high-quality reads from Illumina sequencing data based on recommendations from Minoche et al. (69).

Individual metagenomic samples were competitively mapped against a concatenated file of the reference genome set using bowtie2 v2.4.1 (70) with the “--very-sensitive,” “--end-to-end,” and “--no-unal” flags. Each successfully mapped read was assigned only to a single genome that provided the closest match. We used Samtools v1.9 (71) to generate BAM files from the read alignment data generated by bowtie2. We then used *anvi-single-profile* to create an Anvi’o single-profile database for each metagenome’s BAM file. Single profiles for each sample were merged for each oral site using *anvi-merge*, thus generating a profile database for each oral site. For each metagenome, we used *anvi-summarize* to extract the mapping metrics of mean depth of coverage and breadth of coverage for reads aligned to each genome. Breadth of genome coverage is defined as the proportion of nucleotides covered at least 1×, and a genome was detected within a metagenome if the breadth of coverage was at least 50%, following recently developed procedures (20, 72). For each metagenome, the relative abundance of each genome was calculated by first averaging the depth of coverage (the number of reads mapped to a nucleotide position) across nucleotide positions in the 2nd and 3rd quartiles (the interquartile range) for the reference genome and then dividing by the total mean depth of coverage in the interquartile range for all reference genomes.

### Functional analysis of detected *Veillonella* genomes across human oral sites.

Based on the detection and abundance profiles from metagenomic mapping, reference genomes that showed clear signs of site specialization were assigned to an oral site. We then used the Anvi’o function *anvi-estimate-metabolism* (parameters: --kegg-output-modes modules) to predict the metabolic capabilities of each of the selected genomes. The function determines which enzymes are present in a genome based on KEGG orthologs (KOs) and calculates the percentage of steps in each metabolic pathway (module) that are present in each genome. We used the default module completion threshold of 0.75, which scores a module as “complete” within a genome when at least 75% of the KOs in a module are present. We then used the Anvi’o script *anvi-compute-metabolic-enrichment* to identify significantly enriched complete modules associated with one set of genomes compared to another. Briefly, the script uses a generalized linear model with a logit link function to obtain enrichment scores and adjusted *q* values for each combination of pairwise group comparisons. We also used the Anvi’o script *anvi-compute-functional-enrichment* to identify significantly enriched functional annotations from the NCBI COG, KEGG, and Pfam databases, independent of metabolic pathway completeness, and associated with genomes from an oral site.

### Data availability.

The raw data used in this study are publicly available at NIH GenBank and RefSeq (https://www.ncbi.nlm.nih.gov/genome/) for genomes and Human Microbiome Project for metagenomes (https://portal.hmpdacc.org/). Code used for analyses is available on GitHub (https://github.com/FatherofEverest/Site-specialization-of-human-oral-Veillonella-species.git).
