# Supplementary material for: Site Specialization of Human Oral Veillonella Species
Source: Microbiol Spectr. 2023 Jan 25;11(1):e04042-22. doi: 10.1128/spectrum.04042-22 (PMC9927086; doi:10.1128/spectrum.04042-22)
Supplement: Supplemental file 2 — Supplemental material. Download spectrum.04042-22-s0002.pdf, PDF file, 7.7 MB [file spectrum.04042-22-s0002.pdf]

SUPPLEMENTAL FIGURES

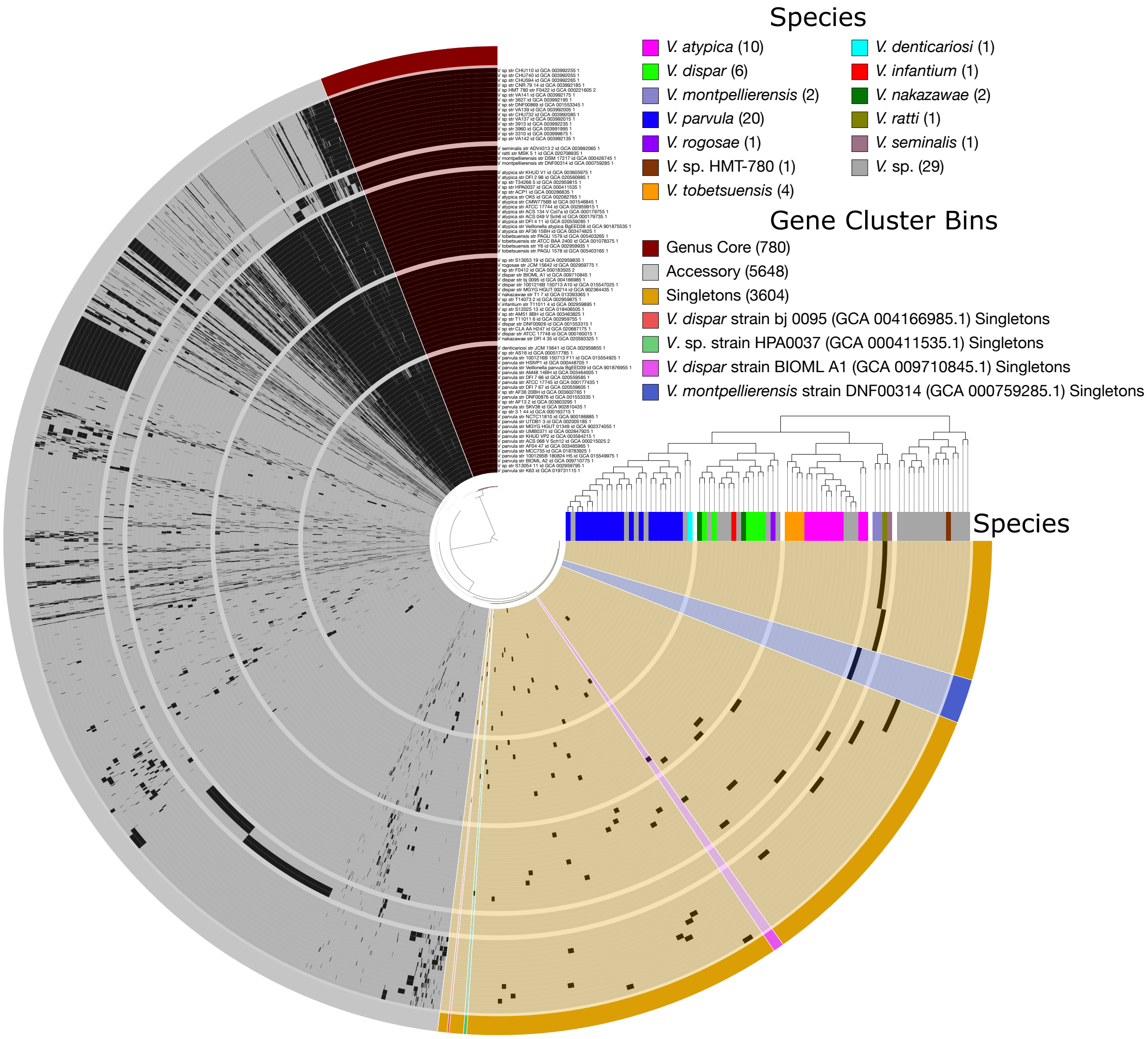

Figure S1. A pangenome of human oral *Veillonella* reference genomes (n = 79) was constructed using a set genomes dereplicated based on a 99% ANI threshold to test the effect of a 98% ANI threshold on a comparative pangenome analysis. Genomes are hierarchically clustered based on gene cluster frequency (i.e., the number of representatives of each gene cluster present in each genome). Gene clusters are arranged based on their presence or absence across the genomes and colored according to their presence in all genomes (core; red; n = 780), a subset of genomes (accessory; gray; n = 5648), or unique to a single genome (singletons; gold; n = 3604). Singleton

gene clusters unique to a genome that was added because of increasing the ANI threshold are colored uniquely.

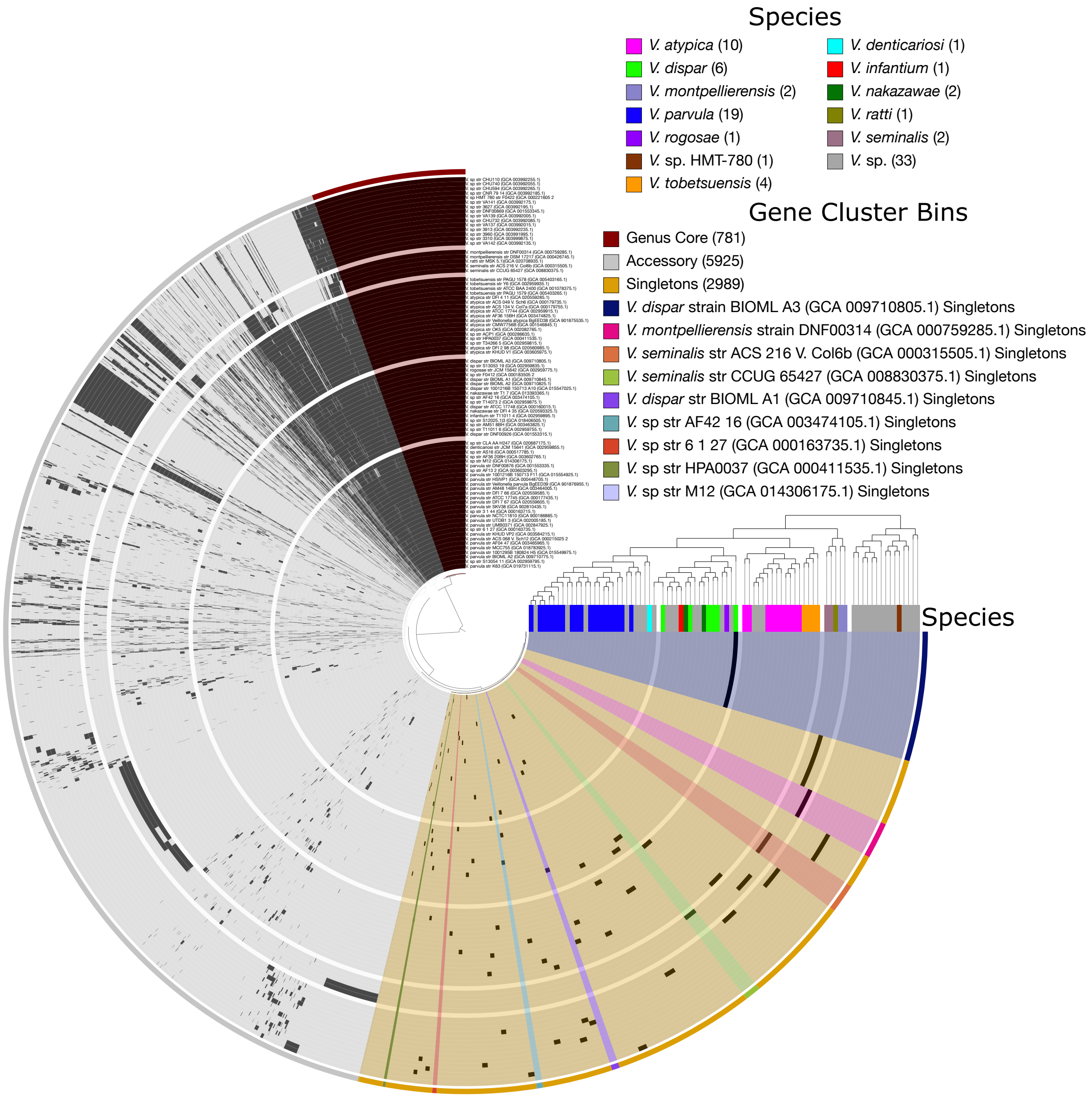

Figure S2. A pangenome of human oral *Veillonella* reference genomes (n = 82) was constructed using a set genomes dereplicated based on a 99.5% ANI threshold to test the effect of a 98% ANI threshold on a comparative pangenome analysis. Genomes and gene clusters are arranged as in Figure S1.

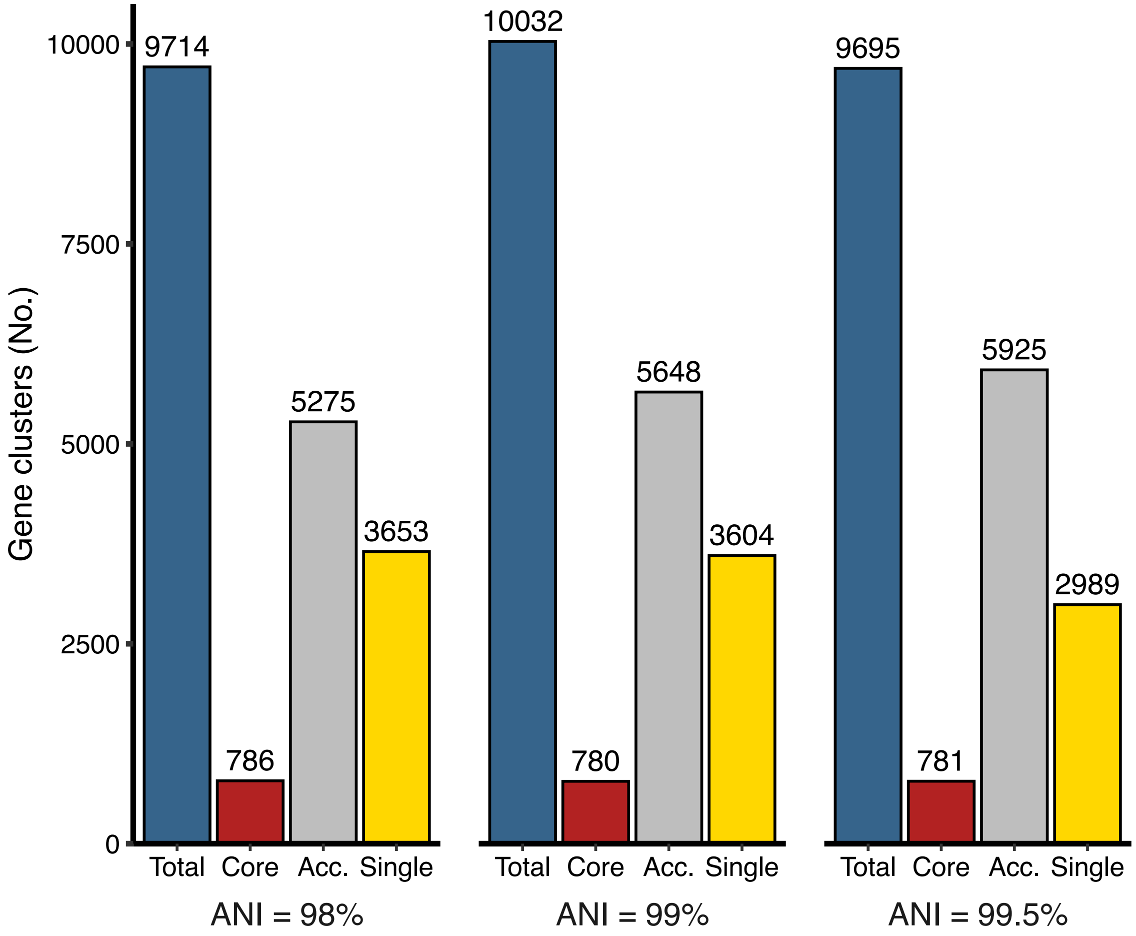

Figure S3. Comparison of the number of gene cluster bins for three pangenomes of human *Veillonella* species that were constructed using a set of genomes dereplicated based on a 98%, 99% or a 99.5% ANI threshold. The distinct gene clusters of each pangenome include core genes that occur in every genome (red), singleton genes that occur in only a single genome (gold) and accessory genes that occur in more than one, but not all genomes (gray).

SUPPLEMENTAL TABLE LEGENDS

Table S1. Metadata for *Veillonella* reference genomes.

Table S2. Whole-genome average nucleotide percent identity for *Veillonella* reference genomes.

Table S3. Metabolic pathway enrichment test results.

Table S4. Results for enrichment of COG20, Pfams and KEGG associations.

Table S5. ANI dereplication test blast results. We analyzed how additional genomes altered accessory gene content by inspecting the singleton gene clusters unique to each of the additional genomes. For each pangenome, we manually binned singleton gene clusters for each of the additional genomes, extracted their amino acid sequences using anvi-get-sequences-for-gene-clusters and blasted each sequence against the NCBI non-redundant protein database (1), which includes protein sequences from GenPept, Swissprot, PIR, PDF, PDB, and NCBI RefSeq.

REFERENCES

1. O'Leary NA, Wright MW, Brister JR, Ciufo S, Haddad D, McVeigh R, Rajput B, Robbertse B, Smith-White B, Ako-Adjei D. 2016. Reference sequence (RefSeq) database at NCBI: current status, taxonomic expansion, and functional annotation. Nucleic Acids Res 44:D733–D745.
